# Reflection on analyzing the long-term quality of life between duodenum-preserving pancreatic head resection and pancreatoduodenectomy

**DOI:** 10.1097/JS9.0000000000001519

**Published:** 2024-05-03

**Authors:** Hanyun Tu, Sufei Yang, Rong Liu

**Affiliations:** a School of Medicine, Jinan University, Guangzhou; b Department of Cardiology, Daping Hospital, Army Medical University, Third Military Medical University, Chongqing; c Sichuan Institute of Product Quality Supervision and Inspection, Chengdu; d Graduate School, The Chinese Academy of Sciences, Kunming, People’s Republic of China

*Dear Editor,*

We wish to express our thoughts regarding the recent publication titled ‘Long-term quality of life between duodenum-preserving pancreatic head resection and pancreatoduodenectomy: a systematic review and meta-analysis’^1^. This study provided valuable insights into resolving the ongoing debate regarding the long-term benefits of duodenum-preserving pancreatic head resection (DPPHR) compared to pancreatoduodenectomy (PD) for treating pancreatic conditions. The authors Yin *et al*. recommend DPPHR over PD for treating benign pancreatic diseases and low-grade malignant tumors since DPPHR’s advantages over PD in terms of safer perioperative outcomes, lower long-term symptom scores, and longer overall survival (OS) times while highlighting no significant difference in quality of life (QOL) scores between the DPPHR and PD groups. However, certain methodological areas warrant a more detailed analysis to understand their implications on the findings’ applicability and reliability.

Subgroup analyses show that follow-up length is a crucial factor in evaluating QOL as delineated that QOL benefits of DPPHR over PD were significantly noted during the follow-up period of 2–7 years, whereas similar QOL outcomes were reported for shorter (<2 years) and longer (>7 years) follow-up durations. This nuanced observation suggests that the optimal timeframe to evaluate the true benefits of certain surgical interventions might lie within a specific follow-up window. Nevertheless, the implications of follow-up duration extend beyond QOL outcomes to encompass other vital aspects such as functional scale, symptom scale, and long-term outcomes. Each of these domains might be differently impacted by the length of follow-up, potentially altering the interpretation of the surgical intervention’s efficacy and safety over time, implying that more subgroup analyses should be employed.

While prioritizing data from the longest follow-up period can highlight the enduring impacts of surgery, acknowledging and analyzing outcomes from all follow-up intervals will provide a more complete picture of the surgical intervention’s effects. The inclusion of data from multiple follow-up periods, as seen in Müller *et al*.’s study^2^, presented QOL outcomes at both 7 and 14 years of follow-up, while only the statistics of 14-year follow-up were included in the meta-analysis, although the 7-year follow-up length is within long-term range.

Moreover, chronic pancreatitis (CP) is a fibroinflammatory disease of the exocrine pancreas, leading to permanent structural damage of the gland and ultimately to the impairment of both the exocrine and endocrine functions of the gland^3^. Concentrating on the onset rate of endocrine and exocrine disorders in the long-term follow-up length, although drawing a result that a non-inferiority of DPPHR compared to PD, is not comprehensive since progressive parenchymal destruction in CP rather than an individual surgical procedure may be mainly responsible for the impairment of pancreatic function. With Diener *et al*.’s study^4^ that some patients with preoperative exocrine or endocrine dysfunction have improved postoperative function, further efforts in the field of CP surgery should consider focusing more on the potential for functional improvement post-surgery.

In addition, randomized controlled trials (RCTs) to analyze OS were completely conducted in Germany, a country with a highly developed healthcare system, which limits the generalizability of the findings. Other limitation factors comprising the applicability of the study’s conclusions to regions with different healthcare infrastructure, surgical expertise, patient demographics, and cultural differences also affect patient-reported outcomes, especially regarding QOL assessments, which are inherently subjective and influenced by local societal norms and expectations^5,6^. More research conducted on a broader geographic scope in the future could help ascertain whether the observed advantages of DPPHR over PD on OS hold across different contexts and improve the universal applicability of research results.

In light of the considerations above, further research employing more rigorous methodological standards, broader geographical inclusion, and a more detailed examination of the long-term follow-up outcomes of DPPHR and PD is crucial. We appreciate the authors’ efforts and hope the authors consider these factors in the ongoing trials.

## Ethical approval

Not applicable.

## Consent

Not applicable.

## Sources of funding

Not applicable.

## Author contribution

H.T.: conceptualization, writing – original draft, and writing – review and editing; S.Y.: writing – original draft and writing – review and editing; R.L.: writing – review and editing and supervision.

## Conflicts of interest disclosure

There are no conflicts of interest.

## Research registration unique identifying number (UIN)

This is a commentary; no UIN is required.

## Guarantor

Rong Liu.

## Data availability statement

Not applicable.

## Provenance and peer review

Our paper was not invited.
